# Advantages of Operating in Certain Cases of Hair-lip at a Very Early Age

**Published:** 1855-01

**Authors:** Henry Smith


Advantages of Operating in certain cases of Hair-lip at a very early age. By Henry Smith, Esq.—It is obvious in the first place, that an infant with hare-lip cannot so readily take in that nourishment which is offered by nature. If, however, the deformity be remedied, the child will be placed, by the aid of surgery, in a much more favorable condition to receive the nutriment afforded by the mother's breast. It is plain, too, that the deformity excites most unpleasant and painful sensations in the mind of the mother and those around her; and, if the source of this anxiety can be removed at once, it is of great importance that it should be accomplished.

A third argument in favor of very early operation for hare-lip consists in the circumstance, that as the growth of the child is very rapid in the first period of life, the lip, with other structures of the body at this time, becomes more fully and fairly developed, and thus, after an operation has been successfully performed, there will be a much less chance of subsequent deformity in the part. But it is in those instances where the hare-lip is complicated with a more or less extensive fissure in the palate that an early operation for the cure of the former is so imperatively demanded, and is attended with some beneficial results; and it is to this point especially I wish to draw attention, because, although in some recent works of surgery an operation at an early period after birth is recommended, (and I may especially allude to the *Practical Surgery* of Prof. Fergusson, and to the *Surgeon's Vade Mecum*, by Dr. Druitt,) the most important reason for such a proceeding is not alluded to. And I now refer to the effect which is produced upon the fissure in the hard palate by the approximation of the edges of the lip. As long as the hare-lip remains in its primitive state, there can be no pressure upon the hard tissues underneath; but, if it be united by the surgeon, a considerable amount of pressure is exerted upon the cleft in the palate; and, in a child aged only a few days or weeks, the bones are so soft and compressible, that they are to a great extent influenced by the pressure which constantly obtains, and in the course of time the fissure becomes either entirely closed or diminished in size to one-third or one-fourth of its original extent.

I have had various opportunities of noticing this effect in instances where a very early operation has been performed for hare-lip, complicated with more or less extensive fissure in the hard palate; and so convinced am I of the importance of performing the operation as soon after birth as possible, that I invariably recommend it. And it has fallen to my lot to be called upon to perform the operation very soon after birth, where there has been, at the same time with the hare-lip, considerable malformation of the palate; and I have been able to notice the result some length of time afterwards. More than three years ago I operated upon an infant only four days old; here there was an extensive fissure extending through the hard palate into the nostril. I had an opportunity of seeing this child only a few days since, and the opening in the front portion of the palate was closed up. In this case the soft palate was extensively cleft, and that still remains open; but the parts altogether are in such a condition that, some years hence, they may be completely closed by staphylorraphy. A few weeks since a little patient was brought to me, on whom I operated at a very early age, two years age. In this instance there was a fissure in the hard palate, and great deformity of the jaw, a portion of which I removed at the time. There is now an admirably developed upper lip, and complete closure of the opening which existed in the palate. In another instance, where I operated at an early period, there was an immense chasm running through both the soft and hard palate into the nostril. I had an opportunity of seeing this patient a few days since, and found that the anterior portion of the cleft was much diminished in size. The operation was done more than a year ago.

Mr. Bateman of Islington, who pays great attention to this matter, operated, three years since, upon an infant only four hours after birth. In this case there was an extensive fissure in the palate. This gentleman kindly showed me this case, and, in reply to my enquiry regarding the effect which the operation had upon the palate, he wrote word the other day that the child had died of whooping-cough last winter, but that its mother remarked that before death the fissure, which had at birth been "so large that she could put her thumb into it, had contracted so much that it would scarcely admit the edge of a sheet of writing paper." About a month since I operated upon an infant only six days old, with perfect success. In this case I was partly induced to perform the operation at this early period because there was a fissure in the hard palate, extending into the nostril. I have little doubt that, in time, if the child lives, the fissure will be completely closed.—*Med. Times and Gazette.*

